# Structural Basis for the Inhibition of SARS-CoV-2 M^pro^ D48N Mutant by Shikonin and PF-07321332

**DOI:** 10.3390/v16010065

**Published:** 2023-12-30

**Authors:** Zhenyu Zhao, Qinyao Zhu, Xuelan Zhou, Wenwen Li, Xiushan Yin, Jian Li

**Affiliations:** 1 College of Pharmaceutical Sciences, Gannan Medical University, Ganzhou 341000, China; 15729159685@163.com (Z.Z.); zhouxuelan0125@163.com (X.Z.); l18679629911@163.com (W.L.); 2 Applied Biology Laboratory, College of Pharmaceutical and Biological Engineering, Shenyang University of Chemical Technology, Shenyang 110142, China; zhuqinyao0815@163.com

**Keywords:** SARS-CoV-2, mutant, main protease, shikonin, PF-07321332, M^pro^ inhibitor

## Abstract

Preventing the spread of SARS-CoV-2 and its variants is crucial in the fight against COVID-19. Inhibition of the main protease (M^pro^) of SARS-CoV-2 is the key to disrupting viral replication, making M^pro^ a promising target for therapy. PF-07321332 and shikonin have been identified as effective broad-spectrum inhibitors of SARS-CoV-2 M^pro^. The crystal structures of SARS-CoV-2 M^pro^ bound to PF-07321332 and shikonin have been resolved in previous studies. However, the exact mechanism regarding how SARS-CoV-2 M^pro^ mutants impact their binding modes largely remains to be investigated. In this study, we expressed a SARS-CoV-2 M^pro^ mutant, carrying the D48N substitution, representing a class of mutations located near the active sites of M^pro^. The crystal structures of M^pro^ D48N in complex with PF-07321332 and shikonin were solved. A detailed analysis of the interactions between M^pro^ D48N and two inhibitors provides key insights into the binding pattern and its structural determinants. Further, the binding patterns of the two inhibitors to M^pro^ D48N mutant and wild-type M^pro^ were compared in detail. This study illustrates the possible conformational changes when the M^pro^ D48N mutant is bound to inhibitors. Structural insights derived from this study will inform the development of new drugs against novel coronaviruses.

## 1. Introduction

SARS-CoV-2 has rapidly spread worldwide since the 2019 outbreak [1]. As of 15 December 2023, COVID-19 has caused 772 million confirmed cases and more than 6.98 million deaths globally “https://covid19.who.int/ (accessed on 15 December 2023)”. We have made preliminary progress in the long-term prevention, control, and management of the virus, but variants of SARS-CoV-2 still pose a threat to human health [2,3]. There are still a lot of problems to be addressed. Infection with COVID-19 results in respiratory infections leading to severe cytokine storm syndrome. In severe COVID-19 cases, cytokine storms can even be life-threatening [4,5]. Current strategies for SARS-CoV-2 focus on how to avoid severe cytokine storms and inhibit viral replication. Since the release of the SARS-CoV-2 genome sequence, many pharmaceutical companies have invested in the development of a SARS-CoV-2 vaccine to curb its spread and infection. Two of these mRNA-based vaccines, Pfizer-BioNTech COVID-19 Vaccine COMIRNATY^®^ and Moderna COVID-19 Vaccine Spikevax^®^ have been approved by the FDA and widely used [6,7]. However, the primary role of the vaccines focuses on preventing viral infections and untimely death. The development of targeted antiviral drugs is urgently needed to treat patients who are already infected. Clearly, combining an anti-viral vaccine with anti-viral drugs is the best approach to combat COVID-19 [8].

The genomic RNA (gRNA) of SARS-CoV-2 contains 14 open reading frames (ORF) [9]. The two major ORF1a and ORF1b are translated into the polyproteins pp1a and pp1ab, which are processed by viral proteases to produce nonstructural proteins (Nsps) [10,11]. Nsp5 is a 33 kDa cysteine protease, also known as main protease (M^pro^), or 3C-like protease (3CLpro). M^pro^ of SARS-CoV-2 produces 12 functional proteins that mediate viral replication and transcription and are highly conserved among several common coronaviruses [12]. Inhibitors targeting M^pro^ would successfully block viral replication, making M^pro^ an attractive target for SARS-CoV-2.

The oral antiviral drug Paxlovid^TM^ has been developed by Pfizer and approved by the FDA for the treatment of COVID-19. The active ingredient of Paxlovid^TM^, PF-07321332, is a potent M^pro^ inhibitor [13,14]. During the clinical trial, Paxlovid reduced the risk of hospitalization or death by 89.1% in high-risk nonhospitalized adults who suffered from COVID-19 [15]. The success of PF-07321332 confirms the potential of M^pro^ as an anti-SARS-CoV-2 target and many M^pro^ inhibitors have been identified thereafter. PF-07817883 (NCT05580003) has now advanced to phase II clinical trials “https://clinicaltrials.gov/ (accessed on 15 December 2023)”. This drug candidate was developed based on the scaffold of PF-07321332, resulting in improved oral absorption and no need for ritonavir to extend blood levels. STI-1558 (NCT05716425), developed by ACEA Pharmaceutical Co., Ltd., Hangzhou, China, has entered phase III clinical trials and is another promising drug candidate “https://clinicaltrials.gov/ (accessed on 15 December 2023)”.

In addition to developing new drugs targeting M^pro^ of SARS-CoV-2, already known compounds have also been evaluated for their inhibitory activity against SARS-CoV-2 M^pro^ using high-throughput screening. Early in the pandemic outbreak, when there was a lack of drugs, researchers tried to use traditional Chinese herbal medicine to alleviate the symptoms of those infected, with exciting success being achieved. However, most of them remain to be studied as therapeutic agents against the SARS-CoV-2 [16]. Shikonin is a phytochemical found in Lithospermum erythrorhizon root and is known for its bioactivity against cancer, oxidative stress, and inflammation [17]. Previously, our lab solved the crystal structure of SARS-CoV-2 M^pro^ bound to shikonin and clarified the molecular basis for the interaction. Together with other studies, our data suggest that shikonin is a promising M^pro^ inhibitor for the development of anti-COVID-19 drugs [18,19].

The threat of the SARS-CoV-2 is largely generated by the high mutation rate [20]. Recently, multiple mutations have been identified in the main proteases of emerging SARS-CoV-2 variants [21]. This paper focuses on the interaction between SARS-CoV-2 M^pro^ D48N mutant and two inhibitors (shikonin and PF-07321332) and explores the effects of D48N mutation of M^pro^ on the binding of these two compounds via comparison with the interaction between inhibitors and M^pro^ wild-type. The SARS-CoV-2 M^pro^ D48N mutant possesses a single amino acid substitution in the residue located near the M^pro^ active site and has been identified in Alpha, Beta, Gamma, Delta, and Omicron variants as well as recently emerged Omicron XBB subvariants, as documented in the Global Initiative on Sharing All Influenza Data (GISAID). The crystal structures of shikonin or PF-07321332 in complex with SARS-CoV-2 M^pro^ D48N mutant were solved to reveal the structural basis for their interaction. The results could help to develop more effective drugs to counteract viral infections caused by SARS-CoV-2 as well as its variants.

## 2. Materials and Methods

### 2.1. Expression and Purification of SARS-CoV-2 M^pro^ D48N Mutant

SARS-CoV-2 M^pro^ D48N mutant was expressed and purified as described previously [22]. It is synthesized by codon optimization technique and cloned into the pET-28a vector; the n-terminal is fused with the 6 × His tag. Briefly, the pET-28a-M^pro^ D48N-HisTEV recombinant plasmid was transformed into competent *Escherichia coli* (*E. coli*) Rosetta DE3 cells. It was cultured in LB to an optical density (OD_600_) value of 0.6–0.8 at 37 °C, 200 rpm. Then 500 µmol/L of isopropyl-β-d-thiogalactopyranoside (IPTG) was used to induce the production of SARS-CoV-2 M^pro^ D48N mutant at 18 °C and 200 rpm for 20 h. Cell pellets were collected by centrifugation (6000 rpm, 15 min) resuspended with lysis buffer, and then lysed by sonication. Cell pellets were removed by centrifugation (6000 rpm, 15 min). Immobilized metal affinity chromatography followed by imidazole gradient treatment was used to elute target proteins. The eluted protein was concentrated and loaded to a SuperdexTM 200 Increase 10/300 GLcolumn (GE Healthcare, Chicago, IL, USA) preequilibrated with buffer containing 25 mM HEPES, pH 7.5, 5mM DTT, 300 mM NaCl, and 10% glycerol. High-purity M^pro^ D48N mutant was concentrated in concentrators (Millipore, Burlington, MA, USA) after adding TEV protease to remove the N-terminal His tag.

### 2.2. Enzymatic Inhibition Assays

Commercially available fluorescent substrates as donor and quencher pairs were purchased. The Fluorescence resonance energy transfer (FRET)-based enzyme assay was performed in a 384-well microtiter plate according to a conventional protocol to test the inhibitory activities of shikonin and PF-07321332 against SARS-CoV-2-M^pro^ D48N mutant. First, shikonin and PF-07321332 were separately dissolved in DMSO and diluted to various concentrations. Further, 1 mL of M^pro^ D48N mutant (200 nM) was incubated with different concentrations of both inhibitors in reaction buffer (50 mM Tris 7.3, 150 mM NaCl, 1 mM EDTA) for 30 min at room temperature. FRET substrate was added to the reaction system at the end of the incubation and the reaction was continuously detected for 20 min to record luminescence. The initial velocity was plotted against various concentrations of shikonin and PF-07321332 to calculate IC_50_ values, using the dose-response curve in GraphPad Prism software, version 10.1.2. Three independent experiments were performed.

### 2.3. Crystallization of M^pro^ D48N-Shikonin and M^pro^ D48N-FP07321332 Complexes

The SARS-CoV-2 M^pro^ D48N mutant was concentrated at 5 mg/mL using an Amicon Ultra-15 concentrator (Millipore). Next, thoroughly mix the concentrated M^pro^ D48N mutant with shikonin or PF-07321332 molecules at a molar ratio of 1:5 and incubate at 0 °C for 30 min for bonding. After a few days, both complexes successfully crystallized at 20 °C by the hanging drop vapor diffusion technique. The crystallization condition for the SARS-CoV-2 M^pro^ D48N mutant-shikonin complex was 0.16 M Na_2_SO_4_, 20% wt/vol PEG 3350. Meanwhile, the crystals of SARS-CoV-2 M^pro^ D48N mutant in complex with PF-07321332 were grown in a storage solution containing 0.1 M HEPES pH 7.4, 10% isopropyl alcohol, and 20% wt/vol PEG 40000.

### 2.4. Data Collection, Structure Determination, and Refinement

Crystals of M^pro^ D48N mutant-shikonin and M^pro^ D48N mutant-PF-07321332 complexes were cryoprotected by immersion in a crystallization buffer supplemented with 20% glycerol and then flash-cooled in liquid nitrogen to collect better X-ray data. All the X-ray diffraction data were collected at 100K at BL10U2 of the Shanghai Synchrotron Radiation Facility (SSRF). All the collected data were processed by the HKL 2000 software and complex structures were determined by molecular replacement methods via the Phaser program. Coot and Phenix were used for atomic modeling and further refinement of the model to achieve optimal resolution. The complete data collection and statistics for the final refinement are shown in Table 1. The coordinates and factors of the M^pro^ D48N-shikonin and M^pro^ D48N-PF-07321332 structures have been deposited in the Protein Data Bank (PDB) under accession numbers 8WUR and 7XB4, respectively.

## 3. Results

### 3.1. Structure Determination of M^pro^ D48N-PF-07321332 and M^pro^ D48N-Shikonin Complexes

**Figure 1:**
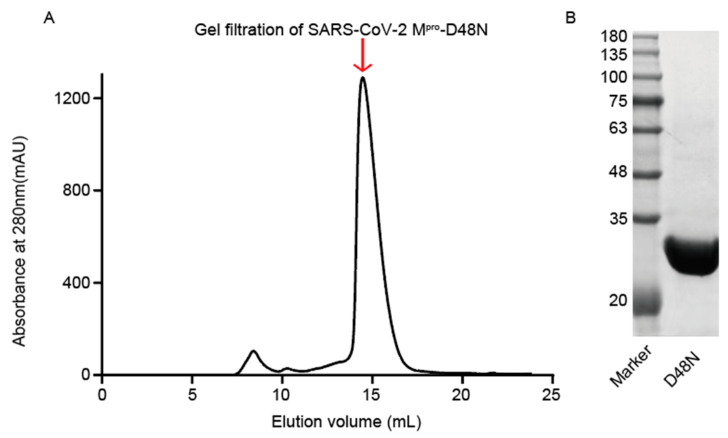
Purification of SARS-CoV-2 M^pro^ D48N mutant. (**A**) Gel filtration was employed to purify the SARS-CoV-2 M^pro^ D48N mutant. A Superdex 200 Increase 10/300GL column was used. (**B**) SDS-PAGE profile of fractions collected during purification of SARS-CoV-2 M^pro^ D48N.

The D48N mutation of SARS-CoV-2 M^pro^ is located on a residue near the M^pro^ active site [21]. The methods described previously were used to express and purify the M^pro^ D48N mutant. The elution volume and molecular weight of the M^pro^ D48N mutant was 33.5 KDa (Figure 1A), which is consistent with previous studies [22].Wild-type M^pro^ has almost the same elution volume and molecular weight as M^pro^ D48N and exhibits similar protein folding efficiency.

In order to clarify the interaction between PF-07321332 or shikonin with M^pro^ D48N, the structures of M^pro^ D48N-PF-07321332 and M^pro^ D48N-shikonin were determined using a co-crystallization method. PF-07321332 and shikonin were each mixed with M^pro^ D48N in a certain ratio to form a complex and to perform co-crystallization. The crystal structures of M^pro^ D48N with the compounds were resolved to 2.07 Å (PF-07321332) and 2.08 Å (shikonin) resolution, respectively. All these complex structures are in space group P2_1_2_1_2_1_. Comparison with previous studies shows that the M^pro^ D48N-shikonin and wild-type M^pro^-shikonin complex structures are in the same space group [18]. However, the wild-type M^pro^-PF-07321332 structure is in P12_1_1 [23], which is different from the M^pro^ D48N-PF-07321332 structure. Data collection and refinement statistics are summarized in Table 1.

### 3.2. Inhibitory Activities of PF-07321332 and Shikonin against SARS-CoV-2-M^pro^ D48N

**Figure 2:**
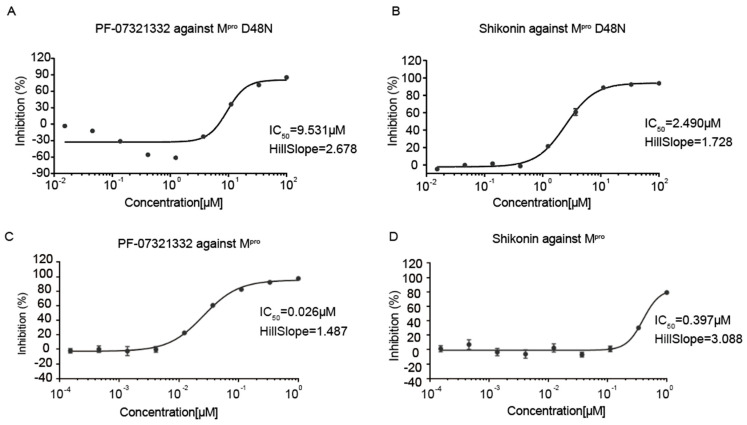
Enzymatic inhibition of SARS-CoV-2 M^pro^ D48N. (**A**) Inhibition of SARS-CoV-2 M^pro^ D48N by PF-07321332. (**B**) Inhibition of SARS-CoV-2 M^pro^ D48N by shikonin. (**C**) Inhibition of SARS-CoV-2 M^pro^ by PF-07321332. (**D**) Inhibition of SARS-CoV-2 M^pro^ by shikonin. Ebselen was used as a positive control. GraphPad Prism software was used to calculate IC_50_.

The inhibitory activities of FP-07321332 and shikonin were determined by fluorescence resonance energy transfer (FRET). The anti-SARS-CoV-2 M^pro^ D48N activity of the shikonin and PF-07321332 was evaluated. Ebselen, a potent covalent inhibitor of M^pro^ [24], was used as a positive control and displayed IC_50_ values of 2.514 μM. In the previous literature [25], the IC_50_ values of Ebselen were at 1.7 ± 0.4 μM, which is comparable to our data. The results showed that PF-07321332 had a potent inhibitory effect against SARS-CoV-2 M^pro^ D48N with an IC_50_ of 9.531 μM (Figure 2A). Shikonin inhibited SARS-CoV-2 M^pro^ D48N with an IC_50_ of 2.49 0μM (Figure 2B), comparable to the positive control. The inhibitory activities of shikonin and PF-07321332 against wild-type M^pro^ were examined using the same method, and the results showed that PF-07321332 had a potent inhibitory effect against wild-type M^pro^ with the IC_50_ value being 0.026 μM (Figure 2C), while shikonin inhibits wild-type SARS-CoV-2 M^pro^ with an IC_50_ of 0.397μM (Figure 2D), both similar with the values determined in our previous studies [16]. These data suggest effective inhibition of shikonin and PF-07321332 against SARS-CoV-2 M^pro^ as well as the M^pro^ D48N mutant.

### 3.3. Inhibitory Mechanism of Shikonin against SARS-CoV-2-M^pro^ D48N

**Figure 3:**
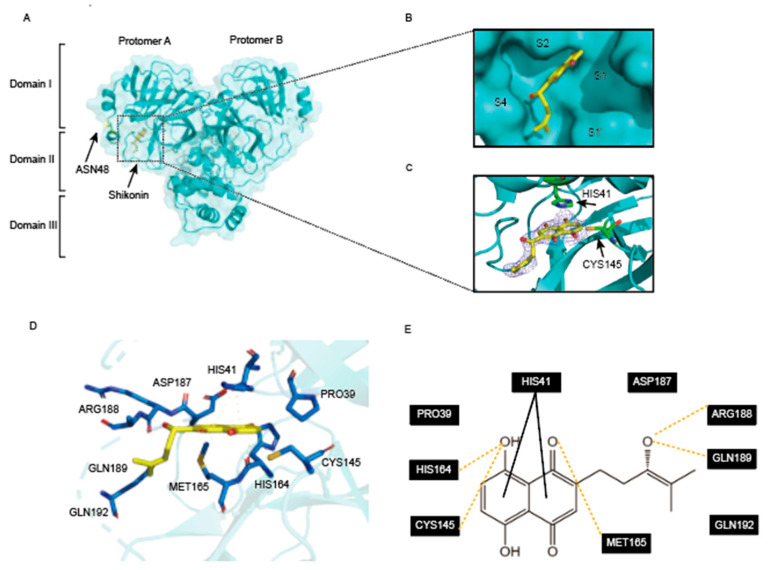
Crystal structure of mutation SARS-CoV-2 M^pro^ D48N in complex with shikonin. (**A**) The overall structure of M^pro^ D48N in complex with shikonin. Protomers A and B are labeled as well as three domains of M^pro^ D48N. The substrate binding pocket is situated within the black dotted box. shikonin is shown as sticks. (**B**) An enlarged view of the substrate-binding pocket. M^pro^ D48N is shown as surface and shikonin is shown as sticks. (**C**) The 2F*o*–F*c* density map contoured at 1.0σ for shikonin. (**D**) The detailed interaction in the M^pro^ D48N-shikonin structure is shown with the residues of SARS-CoV-2 M^pro^-D48N involved in inhibitor binding (within 4 Å), shown as sticks. Hydrogen-bonding interactions are indicated as yellow dashed lines. π-π interaction is indicated as black dashed lines. (**E**) Schematic interaction between shikonin and M^pro^ D48N.

Shikonin and its derivatives are highly secure [26]. No minimal toxicity was observed in in vivo experiments on Wistar rats when shikonin was administered orally at high doses [27]. Shikonin binds non-selectively to M^pro^ in the previous literature and exhibits high inhibitory activity [28]. In order to clarify the mechanism of inhibition of M^pro^ D48N by shikonin, we obtained the complex crystal structure of M^pro^ D48N-shikonin at 2.08 Å resolution. In the solved complex structure, M^pro^ D48N is shown to be a homodimer which is exactly the enzymatic activity form. Each protomer contains three major structural domains. As is shown in Figure 1A, the inhibitor shikonin can be found only in protomer A. Domain I (residues 10 to 99), domain II (residues 100 to 184), and domain III begins at residue 201 and ends at residue 303, which consists of several helices that are linked to domain II and are involved in regulating the dimerization of M^pro^ (Figure 3A). The active pocket of M^pro^ is located between domains I and II and is characterized as a non-canonical duplex of cysteine 145 and histidine 41 (Cys145 and His41) with four binding sites, S1′, S1, S2, and S4. As shown in Figure 3B, shikonin can be found in the protomer A and inserts well into the active site of M^pro^ D48N. Further, the electron density maps of M^pro^D48N-shikonin were extracted. It can be observed that mutation of D48N does not alter the non-covalent inhibition properties (Figure 3C). Next, we analyze the possible interaction of D48N with shikonin (within the 4 Å residues). It was found that shikonin interacts with the catalytic dichotomy (Cys145 and His41). The naphthoquinone of shikonin forms a π-π interaction with His41 at the M^pro^ D48N in the S2 subsite, and to accommodate this force His41 undergoes a dramatic conformational change, also has hydrogen bonding with Cys145. Additionally, shikonin forms hydrogen bonds with Arg188, and Gln189 also has hydrogen bonding interactions with His164 in the S1’pocket (Figure 3D,E). MET165 at the S4 site also underwent a conformational change to form a hydrogen bond with shikonin. All these interactions led to a better occupation of the active site of the M^pro^ D48N by shikonin. This demonstrates the potential of shikonin as a non-covalent inhibitor to block the binding and cleavage of substrates by the SARS-CoV-2 M^pro^ D48N.

### 3.4. Inhibitory Mechanism of PF-07321332 against SARS-CoV-2-D48N M^pro^

**Figure 4:**
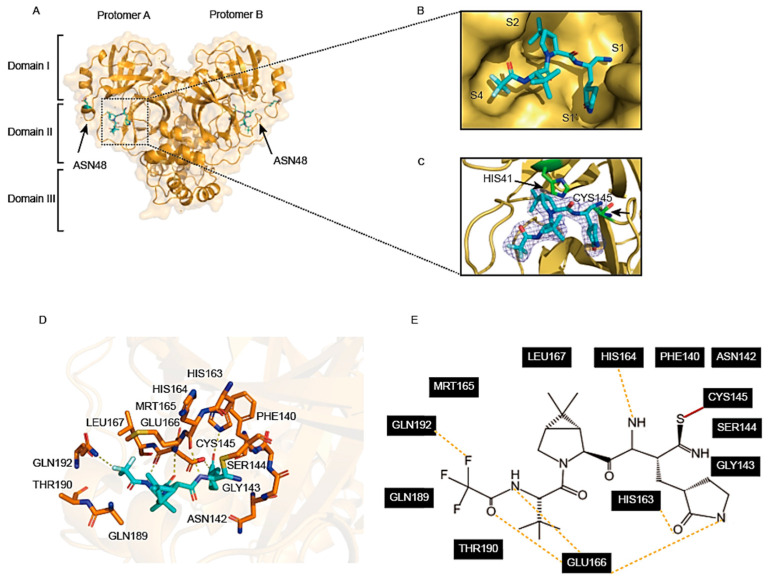
Crystal structure of SARS-CoV-2 M^pro^ D48N mutant in complex with PF-07321332. (**A**) The overall structure of M^pro^ D48N in complex with PF-07321332. Protomers A and B are labeled as well as three domains of M^pro^ D48N. One of the substrate binding pockets is situated within the black dotted box to display. PF-07321332 is shown as sticks. (**B**) An enlarged view of the substrate-binding pocket. M^pro^ D48N is shown as surface and PF-07321332 is shown as sticks. (**C**) A C-S covalent bond forms between Cys145 and the nitrile carbon of PF-07321332. The 2F*o*–F*c* density map contoured at 1.0σ is shown as a blue mesh. (**D**) The detailed interaction in the M^pro^ D48N-PF-07321332 structure is shown with the residues of M^pro^ D48N involved in inhibitor binding (within 3.5 Å), shown as sticks. A covalent bond is shown as a red line. Hydrogen-bonding interactions are indicated as yellow dashed lines. (**E**) Schematic interaction between PF-07321332 and M^pro^ D48N.

Crystals of the M^pro^ D48N-PF-07321332 complex were obtained using the co-crystallisation method. The complex crystal structure of M^pro^-D48N-PF-07321332 was resolved at 2.07 Å resolution. Unlike shikonin, PF-07321332 can be found in both protomers of M^pro^-D48N (Figure 4A). PF-07321332 occupies subsites S1, S2, and S4 of SARS-CoV-2 M^pro^ D48N (Figure 4B). The electron density map shows that the nitrile carbon of PF-07321332 forms a C-S covalent bond with the S atom of Cys145, which is the M^pro^-D48N catalytic residue (Figure 4C). Next, we analyzed the interactions between residues in M^pro^ D48N mutant and PF-07321332 within 3.5 Å. The lactam ring of PF-07321332 can be successfully inserted into the S1 subsite of M^pro^ D48N. The oxygen of the lactam ring forms a hydrogen bond with residue Leu167. The carboxyl group Glu166 forms a hydrogen bond with the nitrogen atom of the lactam ring. Additionally, the oxygen and nitrogen atoms in the trifluoroacetamide of PF-07321332 also have hydrogen bonding interactions with Glu166. The fluorine atom of PF-07321332 forms an additional hydrogen bond by interacting with Gln192. Residue His164 also forms a hydrogen bond with PF-07321332. All these interactions provide PF-07321332 with a stronger anchoring effect and thus better stabilization of the structure (Figure 3D,E). In summary, PF-07321332 occupies the active site of M^pro^ D48N through covalent binding to Cys145 and hydrogen bonding interaction with several conserved residues. It is easy to conclude that PF-07321332 is a highly promising lead compound for the development of antiviral drugs against the SARS-CoV-2 mutant.

### 3.5. Structural Comparison of M^pro^ D48N Mutant to Wild-Type M^pro^ When Bound to Shikonin and PF-07321332

Protein structures of wild-type M^pro^ in complex with shikonin (7CA8) [18] and PF-07321332 (7VLP) [23] were downloaded from the RCSB Protein Data Bank (RCSB PDB). M^pro^-shikonin and M^pro^-PF-07321332 were each compared to the M^pro^ D48N-shikonin and M^pro^ D48N-PF-07321332 structures, respectively. Overall, M^pro^ D48N-PF-07321332 and M^pro^ D48N-shikonin are highly similar in structure to their wild-type complexes with these two inhibitors, with the RMSD values of equivalent Cα atoms being 0.565 Å and 0.223 Å, respectively.

**Figure 5:**
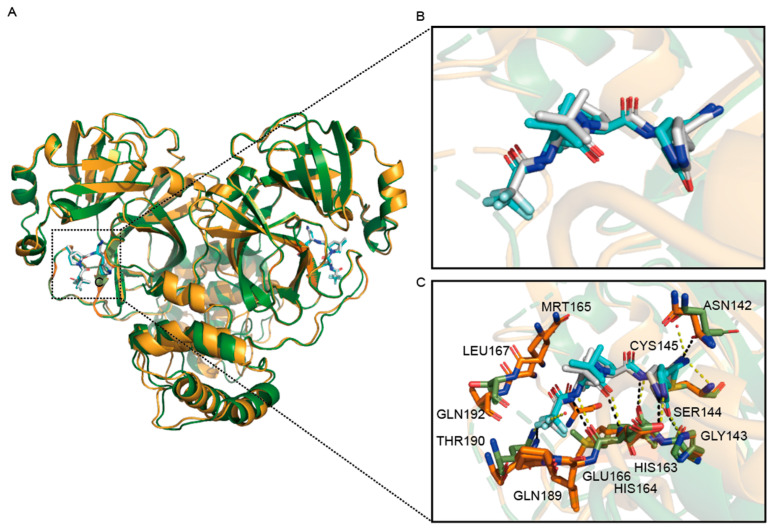
Structural comparison of SARS-CoV-2 M^pro^ D48N-PF-07321332 complexes with the wild-type. (**A**) Overview of structural superposition of wild-type M^pro^-PF-07321332 complex (Green) and M^pro^ D48N-PF-07321332 complex (Orange). M^pro^ and M^pro^ D48N are shown as cartoons, while PF-07321332 are displayed as sticks. (**B**) A zoomed-in view of PF-07321332 binding pocket for M^pro^ (White) and M^pro^ D48N(blue). Water in the wild-type (red) and D48N (orange) complexes are labeled as spheres. (**C**) The detailed interaction in the D48N-PF-07321332 structure is shown with the residues involved in inhibitor binding (within 3.5 Å) displayed as sticks. Red spheres represent water molecules. Hydrogen bond interactions are shown as black (WT) and yellow(D48N) dashed lines.

For PF-07321332, The crystal structure of the wild-type M^pro^-PF-07321332 complex is not significantly different from the M^pro^ D48N-PF-07321332 complex, shown in Figure 5A,B. There are also a few changes in the interaction forces (Figure 5C). The M^pro^ D48N mutant loses the hydrogen bond with the two water molecules that formed to stabilize PF-07321332 in wild-type M^pro^-PF-07321332 structure. Meanwhile, these two structures also differ in hydrogen bonding interactions between Asn142 residue and PF-07321332.

**Figure 6:**
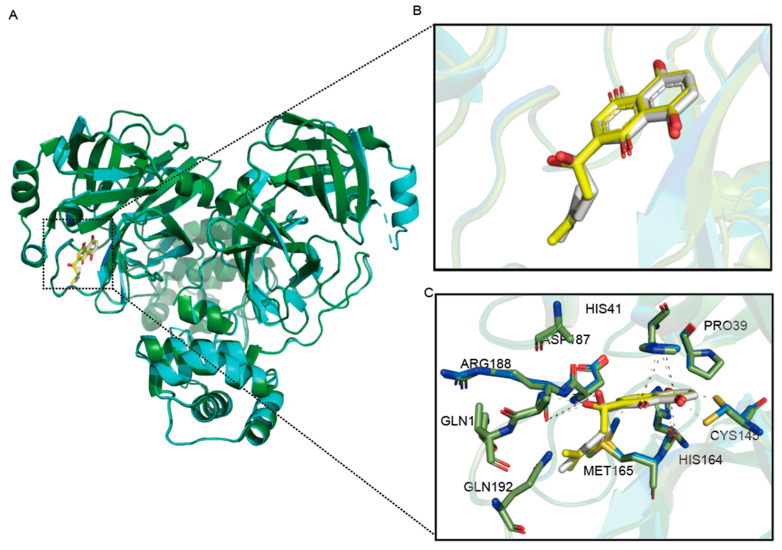
Structural comparison of SARS-CoV-2 M^pro^ D48N-shikonin complexes with the wild-type. (**A**) Overview of structural superposition of wild-type M^pro^-shikonin complex (Green) and M^pro^ D48N-PF-07321332 complex (Blue). M^pro^ and M^pro^ D48N are shown as cartoons, while shikonin are displayed as sticks. (**B**) A zoomed-in view of the shikonin binding pocket for M^pro^ (White) and M^pro^ D48N (Yellow). (**C**) The detailed interaction in the D48N-shikonin structure is shown with the residues involved in inhibitor binding (within 4 Å) displayed as sticks. Hydrogen bond interactions are shown as black (WT) and yellow (D48N) dashed lines.

By comparing the structure of SARS-CoV-2 M^pro^ D48N-PF-07321332 and SARS-CoV-2 M^pro^-PF-07321332 (Figure 6), we can discover these. For shikonin, their interactions have remained largely similar, and the D48N mutation even leads to better binding. The D48N mutation allows shikonin to interact with MET165 at the S4 site by forming a hydrogen bond, suggesting a better fit to the active pocket. These data exclude the possibility of D48N as a drug-resistant mutation and will provide insights for further drug development.

## 4. Discussion

SARS-CoV-2 is posing a continuing economic and public health threat worldwide, and the development of effective anti-viral drugs is an urgent priority in combating this threat. M^pro^, which mediates viral replication, is one of the promising drug targets for SARS-CoV-2. M^pro^ is advantaged in the following: 1. It has no congeners in humans, which implies that its inhibitors do not cause serious harm to humans; 2. Highly conserved among known coronaviruses [29]. However, with the emergence of new variants of SARS-CoV-2, a large number of naturally occurring M^pro^ mutants of SARS-CoV-2 have been reported. In fact, some mutants of M^pro^ have been found to mediate the development of drug resistance in SARS-CoV-2 [30]. It is therefore crucial to understand the effect of structural changes brought about by mutants on inhibitors.

PF-07321332 is the active ingredient of Paxlovid, an oral drug that has been approved for COVID-19, which has been recognized in previous studies as a potent reversible covalent inhibitor of SARS-CoV-2. PF-0732133 exhibits the broad-spectrum inhibition ability of coronaviruses. Another broad-spectrum inhibitor of coronaviruses is the natural product shikonin which exerts its inhibitory effect through non-covalent binding to M^pro^. In this paper, taking SARS-CoV-2 M^pro^ D48N as an example, the conformational changes of PF-07321332 and shikonin binding to the mutant of M^pro^ were investigated.

In this study, the biochemical activity analyses showed that PF-07321332 and shikonin had inhibitory activity against the SARS-CoV-2 M^pro^ D48N. The crystal structures of PF-07321332 and shikonin complexed with M^pro^ D48N were further solved. It was revealed at the molecular level that both PF-07321332 and shikonin were found to interact with the catalytic cysteine of M^pro^ D48N. The M^pro^ D48N is covalently bound to PF-07321332 but from hydrogen bonds with shikonin. In addition, multiple hydrogen bonds are formed with conserved residues within the active site. Shikonin also has a π-π stacking interaction with the His41 residue of the M^pro^ D48N. Both the inhibitors showed different changes in binding to the M^pro^, compared to wild-type M^pro^. However, from the results of enzyme inhibitory activity, there was a large difference in the inhibitory activity of the two inhibitors against D48N and the wild type. However, after analyzing their crystal structures, we could not conclude that D48N is a drug-resistant mutation. The IC_50_ differences are not due to structural differences but may be due to other factors, such as protein stability. These data provide additional referenceable information for drug development and optimization of antivirals against SARS-CoV-2 and provide a theoretical direction for controlling the SARS-CoV-2 variants that emerged at present or in the future.

## 5. Conclusions

Since the main protease of SARS-CoV-2 plays a crucial role in viral replication, it has become one of the most promising targets for the development of antiviral drugs. As new variants of SARS-CoV-2 have been investigated, some intriguing mutants of M^pro^ have been identified. We resolved the crystal structures of two representative broad-spectrum inhibitors, PF-07321332 and shikonin, in complex with the mutant, SARS-CoV-2 M^pro^ D48N. The crystal structures elucidate the mechanism of inhibition of M^pro^ D48N by PF-07321332 and shikonin. These data can be supplemented to develop drugs targeting SARS-CoV-2.

## Figures and Tables

**Table 1:** Statistics for data processing and model refinement of SARS-CoV-2-M^pro^ D48N-Shikinon and SARS-CoV-2-M^pro^ D48N-PF-07321332.

	SARS-CoV-2 M^pro^ D48N PF-07321332	SARS-CoV-2 M^pro^ D48N-Shikonin
PDB code	7XB4	8WUR
**Data collection**		
Synchrotron	SSRF	SSRF
Beam line	BL02U1	BL02U1
Wavelength (Å)	0.97918	0.97918
Space group	P2_1_2_1_2_1_	P2_1_2_1_2_1_
a, b, c (Å)	68.15, 102.66, 103.36	68.05, 102.94, 103.69
α, β, γ (°)	90.00, 90.00, 90.00	90.00, 90.00, 90.00
Total reflections	436,396	543,759
Unique reflections	42,254	44,801
Resolution (Å)	2.07(2.07–2.20)	2.08(2.08–2.19)
R-merge (%)	3.7(86.1)	3.1(106.6)
Mean I/σ (I)	15.7/3.1	18.5/2.7
Completeness (%)	94.4(99.9)	100.0(100.0)
Redundancy	10.3(9.9)	12.1(11.4)
**Refinement**		
Resolution (Å)	56.9–2.07	46.30–2.08
Rwork/Rfree (%)	21.09/24.03	21.07/23.85
Atoms	4514	4568
Mean temperature factor (Å^2^)	36.3	43.5
Bond lengths (Å)	0.008	0.008
Bond angles (°)	0.934	0.969
Preferred	97.28	96.90
Allowed	2.72	3.10
outliers	0	0

## Data Availability

The data presented in this study are available on request from the corresponding author.
